# Changes in the Plasma and Platelet Nitric Oxide Biotransformation Metabolites during Ischemic Stroke—A Dynamic Human LC/MS Metabolomic Study

**DOI:** 10.3390/antiox11050955

**Published:** 2022-05-12

**Authors:** Maciej Bladowski, Ewa Szahidewicz-Krupska, Jerzy Wiśniewski, Paulina Fortuna, Justyna Chojdak-Łukasiewicz, Slawomir Budrewicz, Mariusz Fleszar, Adrian Doroszko

**Affiliations:** 1 Department and Clinic of Internal Medicine, Hypertension and Clinical Oncology, Faculty of Medicine, Wroclaw Medical University, Borowska 213 Str., 50-556 Wroclaw, Poland; maciej.bladowski@umed.wroc.pl (M.B.); ewa.szahidewicz-krupska@umw.edu.pl (E.S.-K.); 2 Department of Medical Biochemistry, Wroclaw Medical University, Chalubinskiego 10 Str., 50-368 Wroclaw, Poland; jerzy.wisniewski@pwr.edu.pl (J.W.); paulina.fortuna@umw.edu.pl (P.F.); mariusz.fleszar@umw.edu.pl (M.F.); 3 Department of Biochemistry, Molecular Biology and Biotechnology, Wroclaw University of Science and Technology, Wyspianskiego 27, 50-370 Wroclaw, Poland; 4 Department and Clinic of Neurology, Wroclaw Medical University, Borowska 213 Str., 50-556 Wroclaw, Poland; justyna.chojdak-lukasiewicz@umw.edu.pl (J.C.-Ł.); slawomir.budrewicz@umw.edu.pl (S.B.)

**Keywords:** ischemic stroke, platelet nitric oxide, L-Arginine, asymmetric dimethylarginine (ADMA), acetylsalicylic acid, platelet aggregation

## Abstract

Despite improvement in the management of modifiable cardiovascular risk factors, ischemic stroke remains the leading cause of morbidity and mortality in the adult population. The aim of this study was to analyze the time-dependent dynamic differences in expression of the nitric oxide (NO) metabolic pathway in the platelet and plasma compartment between subjects with and without ischemic stroke. Additionally, the interplay between these parameters and platelet aggregation was investigated. A total of 418 patients in acute phase of non-cardioembolic stroke were investigated. Following the inclusion and exclusion criteria, finally 40 subjects with stroke and 39 demographically matched healthy participants were enrolled. Neurological physical examination, followed by assessment of the platelet and plasma levels of the nitric oxide synthase (NOS) inhibitors, including asymmetric dimethylarginine (ADMA) and symmetric dimethylarginine (SDMA), as well as NOS substrate-L-Arginine were performed dynamically three times within the first 24-h, then on the 3rd and 7th day after the stroke onset, which was compared with the healthy control. The platelet L-Arginine concentration was significantly higher on the 1st and 3rd day of stroke, while the plasma levels were significantly lower on exact days in comparison to the control. The competitive NOS-inhibitors in platelets were stably elevated in stroke subjects, whereas no significant differences in plasma compartment were noted. The arachidonic-acid-induced platelet aggregation was negatively associated with the platelet NOS substrate bioavailability, as assessed by the $\frac{L-Arginine\;}{ADMA}$-ratio on the 3rd and 7th day. Subjects with non-cardioembolic ischemic stroke are characterized by elevated platelet levels of NOS inhibitors. Management of stroke results in increasing the platelet L-Arginine concentration and subsequent NO bioavailability in the platelet compartment.

## 1. Introduction

Cardiovascular disease (CVD) is among the main causes of morbidity and mortality, as reported worldwide annually. Stroke accounts for approximately 1.1 million new incidents in the European Union each year, with estimated further increase of 3% within the next 3 decades [1]. Ischemic stroke can be divided into subtypes based on the pathomechanism of thrombus origin. Cardioembolic stroke, predominantly caused by the thrombus formation in the left atrial appendage (LAA) during atrial fibrillation, is responsible for ca. 20%, while the non-cardioembolic stroke for 80% of all ischemic stroke cases [2,3].

Endothelial dysfunction and decreased nitric oxide (NO) bioavailability play a pivotal role in the pathogenesis of non-cardioembolic stroke. Limited NO action promotes atherosclerotic plaque formation and its rupture, as well as accelerates platelet activation, adhesion, and aggregation [4,5]. Decreased endothelial NO bioavailability leads to subsequent increase in the expression of adhesive molecules on the platelet surface, such as P-selectin (CD62P), CD40 ligand (CD40L), and ICAM-1, which promote platelet adhesion to the vascular wall and initiate thrombus formation. Activated platelets secrete thromboxane A2, adenosine diphosphate (ADP), and other platelet-derived soluble mediators, which subsequently trigger thrombus growth in a positive feedback loop [6]. Numerous studies have already confirmed that adequate management of cardiovascular risk factors, including hypertension, diabetes mellitus, dyslipidemia, as well as smoking cessation, led to limiting the ischemic stroke burden by increase in endothelial NO bioavailability and restoration of the endothelial vasodilatory function [7].

Interestingly, some new data suggests that not only endothelium, but also platelets are capable of synthesizing the nitric oxide to autoregulate own function. Radziwon-Balicka et al. have recently discovered two separate platelet subpopulations: with or without the ability to synthesize nitric oxide (eNOS-positive or eNOS-negative platelets, respectively). According to the authors, the eNOS negative platelets are the first to form a thrombus on the damaged endothelium, while the eNOS-positive platelets function is to limit the thrombus growth [8]. Furthermore, the authors hypothesize that platelet derived nitric oxide (PDNO) might be a key negative-feedback regulator of thrombus formation in numerous pathological conditions, including acute phase of ischemic stroke.

**Figure 1:**
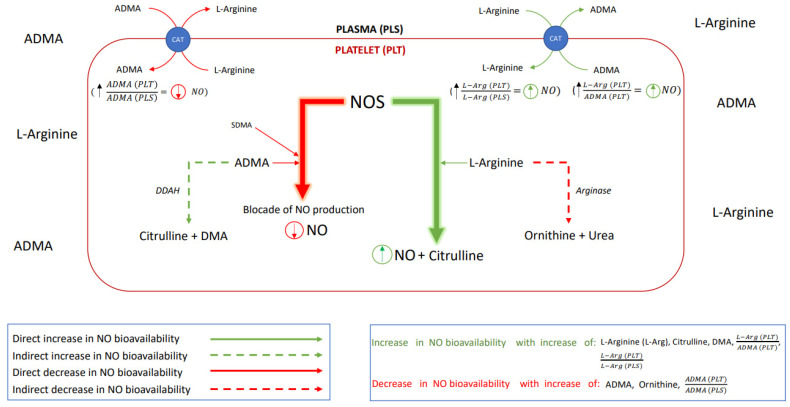
A schematic presentation of the nitric oxide biotransformation. *Abbreviations:* nitric oxide synthase (NOS), nitric oxide (NO), asymmetric dimethylarginine (ADMA), symmetric dimethylarginine (SDMA), dimethylarginine dimethylaminohydrolase (DDAH, an enzyme responsible for ADMA degradation), citrulline, dimethylamine (DMA), L-Arginine (L-Arg), arginase (enzyme responsible for L-Arg degradation), ornithine, urea (product of the L-Arg degradation), cationic-amino acid transporter (CAT, transmembrane L-arginine and ADMA transporter), $\frac{L-Arg\;in\;platelets\;\left(PLT\right)}{L-Arg\;in\;plasma\left(PLS\right)}$ (comparison of nitric oxide bioavailability in platelet vs. in plasma), $\frac{ADMA\;\left(PLT\right)}{ADMA\;\left(PLS\right)}\;$ – (potential for inhibition of NO production in platelet vs. in plasma), $\frac{L-Arg\;\left(PLT\right)}{ADMA\;\left(PLT\right)}$ (potential for the platelet NO synthesis).

Hence, the aim of this study was to verify if alterations in the platelet or plasma NO homeostasis appear in the acute phase of stroke and whether the onset of antiplatelet management restores the nitric oxide biotransformation homeostasis. The effect of the platelet to plasma NO-balance on the platelet aggregation was investigated by analyzing the L-Arginine (L-Arg)—a substrate for NO synthesis; asymmetric dimethylarginine (ADMA) and symmetric dimethylarginine (SDMA)—the competitive NOS inhibitors; then the dimethylamine (DMA)—product of ADMA degradation; citrulline—a product of both L-Arg and ADMA degradation; and ornithine—a product of L-Arg degradation. Schematic presentation of possible interactions between platelet and endothelial nitric oxide biotransformation leading to the maintenance of the NO-dependent platelet functional homeostasis is presented in Figure 1.

## 2. Materials and Methods

### 2.1. Recruitment of Patients

**Figure 2:**
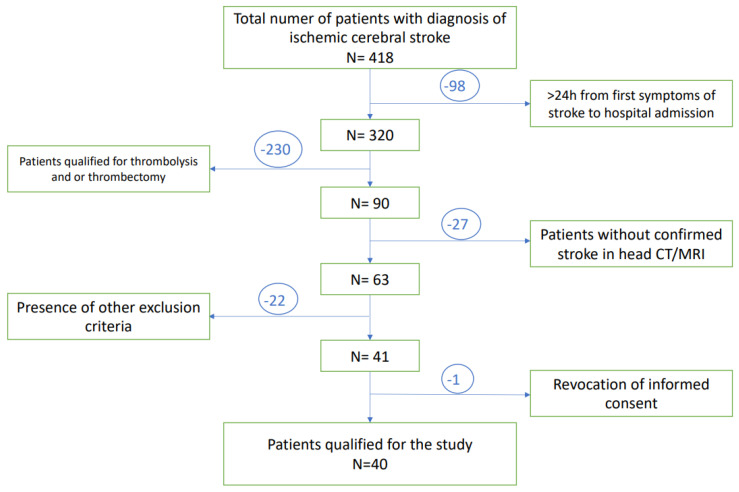
A flow chart of the patients selection.

A total of 418 patients with diagnosis of ischemic stroke, admitted to the University Clinical Hospital in Wroclaw were enrolled in this study. A total of 98 of them were disqualified due to the prolonged time between first neurological symptoms and admission to the hospital (>24 h). Subsequently, 230 patients have undergone thrombolysis and/or thrombectomy and therefore met exclusion criteria. The next 49 patients presented other exclusion criteria. Only one subject revoked informed consent to participate in the study, while none of the patients died during study observation. Finally, a total number of 40 patients at age of 29–80 years with diagnosed acute ischemic stroke were included in the study group. A flow chart presenting the recruitment of the subjects with stroke to the study is presented in Figure 2.

1. The inclusion criteria for the study group were:
  - clinical symptoms of stroke lasting for no longer than 24 h before the hospital admission;
  - diagnosis of ischemic stroke confirmed by neurological examination and/or new cerebral ischemia visualized in the magnetic resonance imaging/computer tomography scan (simultaneously excluding the hemorrhagic stroke);
  - patient signed informed consent to participate in the study.
2. The exclusion criteria for the study group and the control group were:
  - hemorrhagic stroke;
  - thrombolytic treatment or thrombectomy (at present or in the past medical history);
  - history of severe nervous system disease (including previous ischemic or hemorrhagic stroke, neuroinfections, autoimmune, inflammatory, or neurodegenerative diseases);
  - past serious head injuries;
  - atrial fibrillation (previously diagnosed or confirmed during the 72-h ECG monitoring during hospitalization);
  - severe anemia (Hg < 7 g%);
  - thrombocytopenia (platelets < 100,000/μL);
  - ongoing therapies with drugs potentially affecting the obtained results before hospitalization (anticoagulants, antiplatelets drugs, contraceptives, hormone replacement therapy, anti-inflammatory drugs);
  - current infections;
  - active malignancy;
  - chronic inflammatory diseases;
  - chronic kidney disease (eGFR < 45 mL/min/1.73 m^2^);
  - incomplete medical history;
  - inability to provide informed consent.

The control group comprised of 39 volunteers recruited from the hospital outpatient clinic and matched to the study group by demography, similar comorbidities, and undergoing comparable drug therapy before enrolling to the study. Comparison of comorbidities between the control group and study group is shown in Supplementary Table S1, while in the applied treatment is described in Supplementary Table S2.

### 2.2. Study Protocol

**Figure 3:**
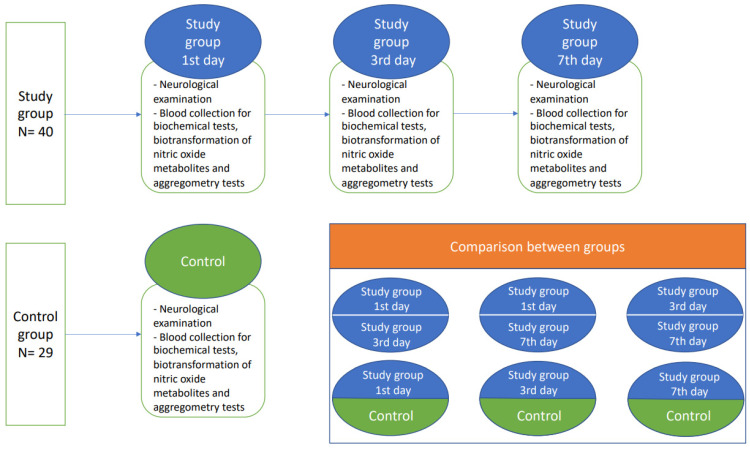
A flow chart of the study protocol.

Patients with diagnosed non-cardioembolic ischemic stroke and initialized acetylsalicylic acid (ASA) treatment (75–150 mg) on the first day of hospitalization were enrolled to the study group after providing written informed consent. Afterwards, the study participants from the stroke group were exanimated thrice: at the time of admission, and on the 3rd and 7th day after ischemic stroke onset. The demographically matched healthy subjects formed the control group, where physical examination, blood collection, and neurological examination was performed once. Subjects from both the study group and control group had to be without any previous history of antiplatelet treatment. ASA treatment was initialized only in the study group (Figure 3).

### 2.3. Blood Collection

The blood for laboratory tests was collected with a single puncture of the antecubital vein, in atraumatic conditions using the S-Monovette set (S-Monovette 10 mL 9NC with tri-sodium citrate at concentration of 0.106 mol/L; S-Monovette 4.9 mL with silicate as clot activator; S-Monovette with 1.6 mg EDTA/mL of blood; Sarstedt AG & Co, Sarstedt, Germany). Whole blood collected in a tube with silicate as an activator of coagulation was centrifuged for 15 min at 1000× *g* in 45 min from its collection. Preserved in the Eppendorf tube, serum was transferred to the accredited university hospital laboratory. The tests were performed using routine certified biochemical methods.

### 2.4. Platelet Preparation for Liquid Chromatography–Mass Spectrometry (LC/MS) Analysis of the Nitric Oxide Metabolic Pathway

The collected whole blood to the S-Monovette tube with trisodium citrate was supplemented with prostacyclin (PGI_2_) at the final target concentration of 0.16 μM and centrifuged for 20 min at 230× *g* at 21 °C to obtain the platelet-rich plasma (PRP). Subsequently, PRP was supplemented with PGI_2_ (with final concentration of 0.8 μM) and centrifuged for 10 min at 1000× *g* at 21 °C. The plasma was discarded, and the platelet pellet was carefully washed three times with 1 mL of the Tyrodes HEPES buffer (134 mM NaCl, 2.9 mM KCl, 1 mM MgCl_2_, 0.34 mM Na_2_HPO_4_, 12 mM NaHCO_3_, 20 mM HEPES, 5 mM Dextrose) pH 7.4. The resulted suspension was immediately analyzed for platelet count and contamination with white blood cells and red blood cells (Sysmex device, Sysmex, Norderstedt, Germany). Samples containing platelets in amounts of 5.0 × 10^8^ cells were preserved for subsequent LC-MS analysis. The samples were obtained by centrifugation of the suspension of known concentration for 5 min, 10,000× *g* at 4 °C, and stored at −80 °C until further analyses [9,10].

### 2.5. Assessment of the Platelet Derived Nitric Oxide Metabolites

Previously prepared platelet samples (PRP), as described in Section 2.4, were thawed on ice. Subsequently, 10 µL of internal standard solution and 1200 µL of cold extraction solution containing methanol, acetonitrile, and water (5:3:2) were added and vortexed (15 min, 1200 rpm, 4 °C). Samples were centrifuged (15 min, 22,000× *g*, 4 °C) and clear supernatants were transferred into new microcentrifuge tubes. Samples were then dried at 50 °C.

Amino acids derivatization was performed using benzoyl chloride (BCl) reagent. Dried samples were dissolved in 100 µL of water and vortexed (5 min, 1200 rpm, 25 °C). Subsequently, 50 µL of borate buffer (0.025 M Na_2_B_4_O_7_·10H_2_O, 1.77 mM NaOH, pH = 9.2), 400 µL of acetonitrile, and 10 µL of 10% BCl in acetonitrile were added and vortexed again (10 min, 1200 rpm, 25 °C). After derivatization, samples were dried at 45 °C using SpeedVac Vacuum Concentrator. Dried samples were reconstituted in 50 µL of 3% of methanol in water and centrifuged (10 min, 15,000× *g*, 4 °C). Supernatant was transferred into chromatographic polypropylene vial with attached 200 µL insert.

Liquid chromatography-mass spectrometry (LC-MS) analysis was performed using SYNAPT G2 Si mass spectrometer coupled with Acquity I-Class UPLC system (Waters, Milford, MA, USA). MS was equipped with electrospray ionization source (ESI). The sprayer voltage, source temperature, desolvation temperature, and desolvation gas flow were set at 0.5 kV, 140 °C, 450 °C, and 900 L/h, respectively. The UPLC system was equipped with cooled sample manager; samples temperature was 8 °C and the injection volume was 2 µL. The Waters BEH Shield C18 column (1.7 µm, 2.1 × 50 mm) was heated to 60 °C. The flow rate was 0.350 mL/min, and the total time of the method was 8 min. The mobile phase solvent A was water with 0.1% formic acid (FA) and solvent B was methanol with 0.1% FA. The following gradient method was used: 0.0 min—3% B, 2.5 min—14% B, 4.6 min—60% B, 4.8 min—90% B, and 6.1 min—3% B. Data acquisition was performed using MassLynx 4.1 software (Waters) for the following ions (*m*/*z*): 237.1239, 243.1339, 263.1090, 267.1382, 279.1457, 286.1897, 307.1770, and 314.2209 for ornithine, D6-ornithine, citrulline, D4-cytrulline, L-Arginine, D7-arginine, ADMA, SDMA, and D7-ADMA, respectively [11].

### 2.6. Measurement of the Plasma Nitric Oxide Metabolites

To obtain plasma, the blood was collected in tubes with EDTA as an anticoagulant (1.6 mg-EDTA/mL blood) and were centrifuged within 30 min after collection at 1000× *g* for 15 min at 4 °C and stored at −20 °C until further analysis. A total of 100 μL of plasma, 50 µL of borate buffer, and 10 µL of internal standard solution (100 µM D7-L-Arginine, 20 µM D7-ADMA, 25 µM D6-DMA, 100 µM D6-ornithine, and 50 µM D4-citrulline) were transferred into 2 mL polypropylene tubes and mixed (1 min, 1200 RPM, 25 °C). Then, 400 µL of acetonitrile and 10 µL of 10% BCl in acetonitrile were added and mixed (10 min, 1200 RPM, 25 °C). Subsequently, samples were centrifuged (7 min, 4 °C, 22,000× *g*) and 100 µL of clear supernatant was diluted four times with water, transferred to chromatographic glass vials, and analyzed. LC-MS analysis was performed using the equipment and methods described above [12].

### 2.7. Measurement of Platelet Aggregation

Platelet function was assessed using the impedance aggregation method in whole blood using the four-channel optical aggregometer (Chrono-log 700, Chrono-Log, Havertown, PA, USA). This method is based on multiple platelet aggregation on the electrodes and changing of the electrical resistance between their two wires. The whole blood was collected to the polypropylene tubes for 10% sodium citrate using the Sarstedt S-Monovette^®^ (Sarstedt Ag & Co., Nümbrecht, Germany) aspiration and vacuum kit. After collection, the tubes were kept at room temperature for a maximum of 90 min before engaging the test. Three different aggregation activators were used: adenosine diphosphate (ADP), arachidonic acid (AA), and collagen. The 1:1 solution of whole blood at room temperature with 0.9% natrium chloride was placed in the test chambers. Then, a certain number of agonists, necessary to obtain appropriate concentrations (0.5 mg/mL for AA, 20 µmol for ADP, 1 µg for collagen), were added to the prepared solution. After 6 min, aggregation curves were recorded, measured, and analysed by dedicated software (Aggrolink^®^, Chrono-Log, PA, USA). The increase in electrical impedance was given in aggregation units.

### 2.8. Statistical Analysis

Statistical analysis was performed using the Statistica 13.3 StatSoft^®^. The presented data is expressed as an arithmetic mean with SEM or median with 1st and 4th quartile if the distribution of variables were not normal. The Mann-Whitney U-test or a student’s t-test, following the Shapiro-Wilk test and Levene’s test as appropriate, were used to assess the significance of differences between the mean values and ANOVA followed by Tukey’s test, or a Friedman test was used when more than two groups were investigated. Spearman test was performed to assess the correlation between nitric biotransformation metabolites and platelet aggregation.

## 3. Results

### 3.1. Baseline Characteristics

The stroke subjects and healthy controls were matched with respect to the age and sex distribution. There were however differences between groups in white blood count (WBC), glucose level, mean platelet volume (MPV), potassium, and thyroid-stimulating hormone (TSH). The baseline demographic and biochemical characteristics of both groups is presented in Table 1.

### 3.2. The Plasma Nitric Oxide Metabolites

**Figure 4:**
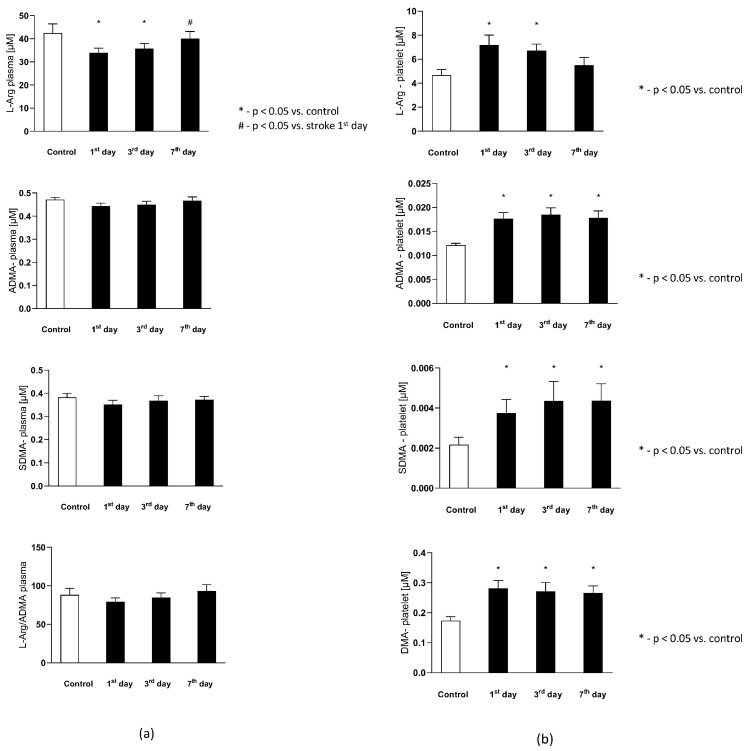

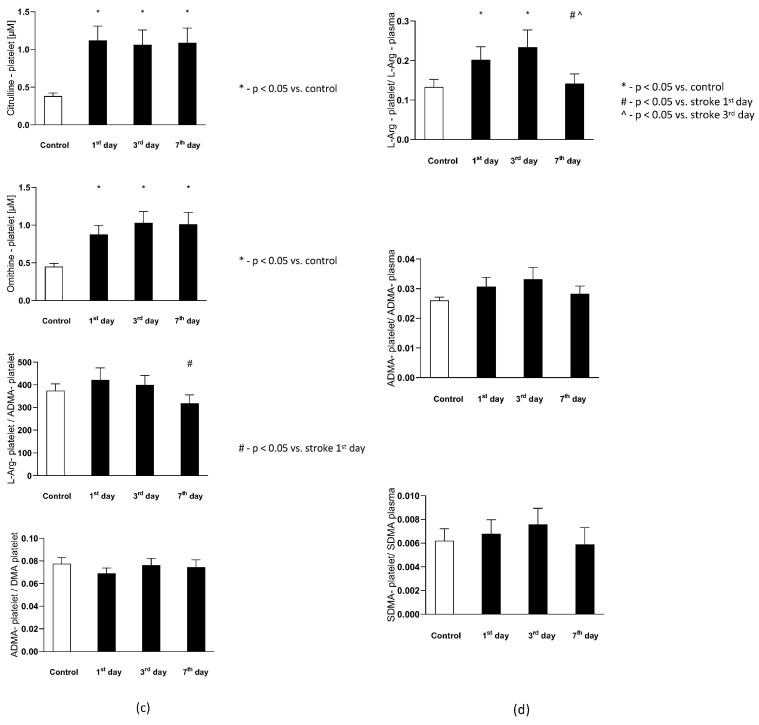

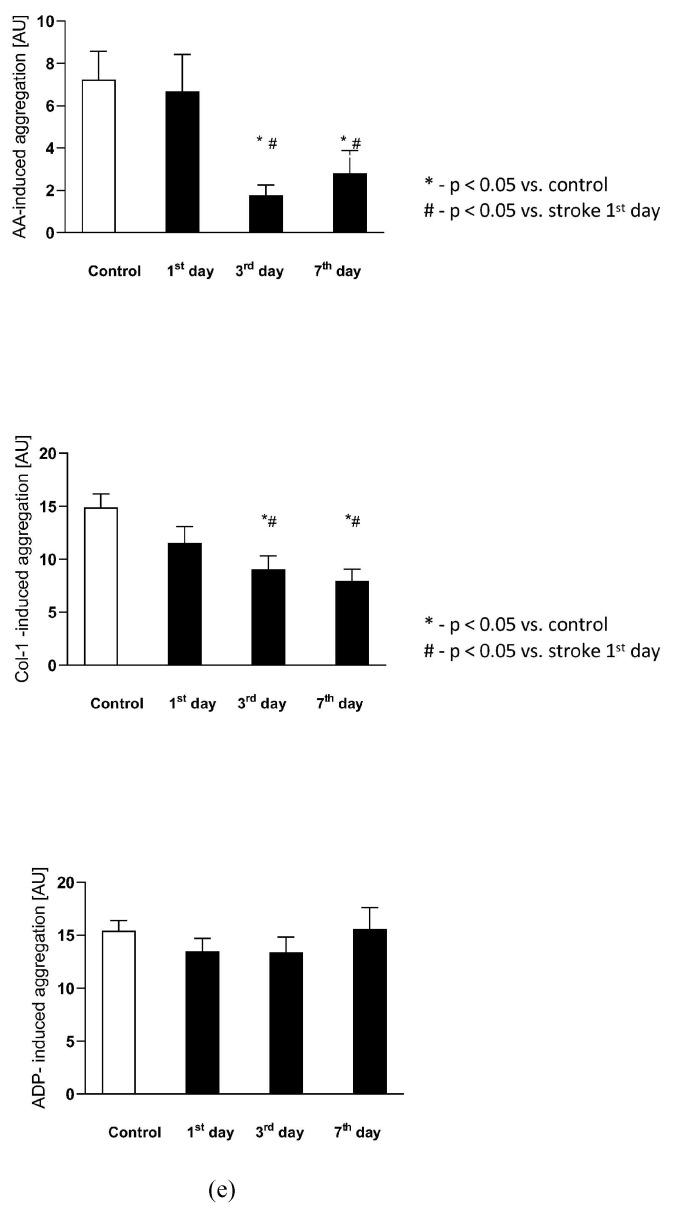
(**a**) The plasma nitric oxide metabolic pathway metabolites in the control group and the study group. *Abbreviations:* Plasma L-Arginine (L-Arg-plasma), the plasma asymmetric dimethylarginine (ADMA-plasma), the plasma symmetric dimethylarginine (SDMA-plasma), L-Arginine to ADMA ratio in plasma (L-Arg/ADMA-plasma). (**b**,**c**) The intraplatelet nitric oxide metabolites in the control group and the study group. *Abbreviations:* L-Arginine in platelets (L-Arginine-platelet), asymmetric dimethylarginine in platelets (ADMA-platelet), symmetric dimethylarginine in platelets (SDMA-platelet), dimethylamine in platelets (DMA-platelet), L-Arginine to asymmetric dimethylarginine ratio in platelets (L-Arginine-platelet/ADMA-platelet), asymmetric dimethylarginine to dimethylamine ratio in platelets (ADMA-platelet/DMA-platelet). (**d**) The balance of the NO biotransformation metabolites between the platelet and plasma compartment. *Abbreviations:* L-Arginine in platelet to L-Arginine in plasma ratio (L-Arg-platelet/L-Arg-plasma), asymmetric dimethylarginine (ADMA), symmetric dimethylarginine (SDMA). (**e**) Platelet aggregation in the control group and the study group. *Abbreviations:* arachidonic acid-induced aggregation (AA-induced aggregation), collagen-1-induced aggregation (COL 1-induced aggregation), adenosine diphosphate- induced aggregation (ADP-induced aggregation), *—*p* < 0.05 vs. control, #—*p* < 0.05 vs. stroke in the first day.

The plasma L-Arginine in the stroke group on the 1st and 3rd day were significantly lower than in the control group, while on the 7th day it reached a level not statistically different from the control group. Plasma ADMA and SDMA levels, together with L-Arginine to ADMA ratio in plasma ($\frac{L-Arg\;\left(PLS\right)}{ADMA\;\left(PLS\right)}$)-reflecting the NOS substrate bioavailability, were not significantly different from the control group (Figure 4a).

### 3.3. Intraplatelet Nitric Oxide Metabolites

The intra-platelet nitric oxide metabolites in both groups are presented in Figure 4b. Six out of eight evaluated nitric oxide metabolites in platelets were found to be significantly higher in subjects with stroke. The platelet L-Arginine on th 1st and 3rd day was greater in the study group, while on the 7th day its level decreased to the concentrations observed in the control group. The ADMA, SDMA, DMA, citrulline, and ornithine concentration on each of the testing days were elevated in the stroke group, in comparison to the healthy individuals. The platelet L-Arginine to ADMA ratio ($\frac{L-Arg\;\left(PLT\right)}{ADMA\;\left(PLT\right)\;}$) decreased consecutively from the 1st to 7th day in the stroke group, but it did not reach a statistical significance in comparison to the control group. Finally, the ADMA to DMA ratio in platelets ($\frac{ADMA\;\left(PLT\right)}{DMA\;\left(PLT\right)}$) from the stroke group was not significantly different from the control group on any of the analyzed days.

### 3.4. The Balance of the NO Biotransformation Metabolites between the Platelet and Plasma Compartment in Subjects with Stroke and in the Control Group

The balance of the NO biotransformation metabolites between the platelet and plasma compartment in both groups is presented in Figure 4d. Only the platelet to plasma of L-Arginine ratio ($\frac{L-Arg\;\left(PLT\right)}{L-Arg\;\left(PLS\right)}$) showed significant difference between groups. On the 1st and 3rd day it was higher in the stroke group, while on the 7th day it was similar as in the control group. There were no differences between groups in the platelet to plasma ADMA ($\frac{ADMA\;\left(PLT\right)}{ADMA\;\left(PLS\right)}$) and SDMA ($\frac{SDMA\;\left(PLT\right)}{SDMA\;\left(PLS\right)}$) ratios in any of the analyzed days.

### 3.5. Platelet Aggregation

There were no differences in the arachidonic acid (AA), collagen-1 at 1 μg/mL concentration (Col-1), nor in the adenosine diphosphate (ADP)-induced platelet aggregation between the control group and stroke subjects on the 1st day after the stroke onset. The AA-induced aggregation significantly decreased on the 3rd and 7th day in comparison to the control group and to the stroke group on the 1st day, reflecting the beginning of ASA treatment. Similarly, Col-1-induced aggregation significantly decreased on the 3rd and 7th day in comparison to the control group and to the 1st day of stroke. The ADP-induced aggregation did not change following ASA treatment on any of the analyzed days. The platelet aggregations in both groups are presented in Figure 4e.

### 3.6. The Correlation between NO Biotransformation Metabolites and Platelet Aggregation

The arachidonic acid-induced aggregation was negatively correlated with the L-Arginine concentration in platelets on the 3rd day, whereas the platelet NO-bioavailability reflected by the $\frac{L-Arg\;\left(PLT\right)}{ADMA\;\left(PLT\right)}$ ratio was both on the 3rd and 7th day. The $\frac{ADMA\;\left(PLT\right)}{ADMA\;\left(PLS\right)}$ ratio was positively associated with AA-induced aggregation only on the 7th day.

The collagen-1-induced aggregation was negatively associated with the platelet NOS substrate bioavailability reflected by the $\frac{L-Arg\;\left(PLT\right)}{L-Arg\;\left(PLS\right)}$ and $\frac{L-Arg\;\left(PLT\right)}{ADMA\;\left(PLT\right)}$ ratios on the 7th day. Interestingly, at the same time, a positive association with the NOS inhibitor bioavailability expressed as the $\frac{ADMA\;\left(PLT\right)}{ADMA\;\left(PLS\right)}$ ratio was noted. Col-1-induced aggregation was also negatively correlated with the platelet citrulline concentration on the 3rd day, and positively with ornithine level on the 7th day.

The ADP-induced aggregation was positively correlated with the ADMA platelet to plasma ratio but only on the first day following the stroke onset. The ADP-induced aggregation was also significantly associated with the $\frac{L-Arg\;\left(PLT\right)}{L-Arg\;\left(PLS\right)}$ ratio on the 3rd day. The rest of the analyzed NO metabolites and their ratios were not correlated with the AA--, Col-1-, nor with ADP-induced aggregation. The correlations between NO biotransformation metabolites and AA-, Col-1-, and ADP- induced aggregation are shown in Supplementary Material: Table S3a–c.

## 4. Discussion

This is the first study to analyze the dynamic, time-dependent changes in the intraplatelet and plasma expression of selected nitric oxide metabolites during acute phase of human ischemic stroke. Furthermore, we are the first to assess the correlation between platelet aggregation and nitric oxide metabolites from these two compartments in stroke patients.

### 4.1. Nitric Oxide Biotransformation Metabolites in Plasma

Our study showed initially decreased plasma level of L-Arginine during ischemic stroke with its gradual increase to the control level within one week following the stroke onset. Other authors have already shown that the lower concentration of L-Arg and the longer time it takes for L-Arg to reach the control level, the greater magnitude of neurologic deterioration and the poorer outcome in ischemic stroke patients is observed [13,14]. Up to date, the overall effect of the plasma nitric oxide during the first days after stroke onset remains uncertain. Serrano-Ponz et al. documented that an increase in the plasma NO bioavailability detected within the first two days of stroke was associated with the lower National Institutes of Health Stroke Scale score (NIHSS), as assessed both on the 7th day and after 3 months following the ischemic event. Noteworthy, a steep increase in the plasma NO bioavailability detected from day 2 to day 7 was associated with an increase in infarct size and, as consequence, in the magnitude of neurological deterioration [15]. This unambiguous, Janus-like effect of the NO could be related to the source of its production. The constitutional endothelial NOS (eNOS) isoform is activated at the beginning of the ischemic stroke, facilitating vasodilatation, inhibition of platelet aggregation, and induction of angiogenesis [16]. Noteworthy, the activation of inducible NOS isoform (iNOS) is initialized a few days after the stroke onset and is considered to damage the surrounding tissue due to the participation in inflammation and unregulated peroxynitrite (NOO^−^) production [17,18]. The enhanced oxidative stress during ischemic stroke could also lead to endothelial NO synthase dysfunction (eNOS uncoupling) characterized by production of superoxide instead of NO. The eNOS uncoupling could be a reason for reduced endothelial transport of L-Arginine, increased rate of L-Arginine efflux, and finally gradual increase of plasma L-Arginine [19,20], which was also detected in our study.

We have shown that the plasma ADMA and SDMA concentrations, together with the plasma L-Arginine/ADMA ratios, did not differ between the stroke and control group. In most of the studies, elevation of the NOS inhibitors concentration and decrease in the L-Arginine/ADMA ratio have been associated with endothelial dysfunction being thus well-recognized CVD risk factors [21,22,23]. Nevertheless, significantly increased plasma ADMA and SDMA concentration were not identified in every study on acute ischemic stroke. According to Brouns et al., the inconsistency regarding the relevance of ADMA and SDMA levels might be linked to the stroke severity. Other authors have already shown that the plasma concentrations of NOS inhibitors are associated with greater severity of stroke, as assessed by the NIHSS score [24,25,26]. Noteworthy, our study group consisted mostly of mild to moderately severe stroke cases with no further progression of neurological deficits.

### 4.2. Nitric Oxide Biotransformation Metabolites in Platelets

We have found significantly higher platelet L-Arginine concentration (substrate for endothelial NOS-eNOS) on the first and third day after the onset of stroke compared to the control group. Although it is well-known that platelet aggregation is NO-dependent, only recently Radziwon-Balicka et al. have documented that platelet can produce nitric oxide on their own. According to those authors, platelets can be divided into two groups depending on ability or inability to produce nitric oxygen (eNOS-positive or eNOS-negative platelets, respectively). The eNOS-negative platelets play a key role in a thrombus formation, while the eNOS-positive ones are responsible for limiting the thrombus growth and consecutively for its dissolving by intraplatelet NO production [8,27]. However, to our knowledge, there was no human study conducted yet on changes in the platelet-derived nitric oxide and its metabolites during acute phase of ischemic stroke.

Our study has also shown stably and significantly elevated platelet concentration of ADMA, SDMA, DMA, citrulline, and ornithine in patients with stroke during the 7-day period of observation. The ADMA and SDMA in platelets, as NOS competitive inhibitors, are responsible for decreased platelet derived nitric oxide production. Gawrys et al. have already demonstrated that the intraplatelet ADMA concentration may promote platelet activation in diabetes mellitus [28]. However, in the study by Meirelles et al., increased ADMA level led to enhanced platelet aggregation in both hypertensive and healthy subjects [29]. Stably elevated levels of the intraplatelet NOS-inhibitors (ADMA, SDMA) and their metabolites (including the DMA, ornithine, citrulline) throughout acute stroke event suggest the presence of their increased pro-aggregatory function before the onset of ischemic stroke. This leads to the conclusion that L-Arginine and ADMA from platelets may be important in two different intervals of ischemic stroke course (ADMA before, and L-Arginine after the thrombotic event), nevertheless further studies in this matter are required.

### 4.3. The Balance of the NO Biotransformation Metabolites between the Platelet and Plasma Compartment

NO is a highly reactive molecule and has an ability to diffuse through a cell membrane [30]. However, the nitric oxide biotransformation metabolites must be actively transported between plasma and platelet compartment in both ways via the cationic amino acid transporters (CAT) or y (+) L system [31]. In our study, only the platelet to plasma L-Arginine ratio ($\frac{L-Arg\;\left(PLT\right)}{L-Arg\;\left(PLS\right)}$) was significantly different between the study group and control group. High platelet and low plasma L-Arginine level at the onset of ischemic stroke were followed by the platelet decrease and plasma increase of L-Arg during the 7 days of observation. Mury et al. have already demonstrated reduced plasma levels of L-Arginine, lower nitric oxide synthase activity, and compensatory increase in L-Arginine transmembrane transport from plasma to platelet compartment in patients being at high risk of thrombotic event [32]. Furthermore, Mendes Ribeiro et al. showed decreased plasma L-Arginine concentration and increased platelet capacity for L-Arginine transport in patients with heart failure or chronic kidney disease [31]. It may suggest the presence of the active NO biotransformation metabolites transport between plasma and platelet compartment also in acute phase of ischemic stroke, however direct measurement of such a transport was not conducted in our study.

Moreover, we have not observed significant changes in $\frac{ADMA\;\left(PLT\right)}{ADMA\;\left(PLS\right)}$ and $\frac{SDMA\;\left(PLT\right)}{SDMA\;\left(PLS\right)}$ ratios in our study. Tymyios at al. demonstrated that elevated plasma ADMA level does not alter platelet NO production, while De Meirelles et al. found that plasma ADMA can decrease the intraplatelet NOS activity [29,33]. Inconsistency in the effect of the plasma ADMA concentration on platelet NO production suggests the complexity of mechanisms controlling the PDNO release. The accumulation of NOS inhibitors in thrombocytes could inhibit further plasma to platelet ADMA transport and be a trigger factor for ischemic stroke incidence, however further studies are required [34].

### 4.4. The Correlation between NO Biotransformation Metabolites and Platelet Aggregation

There are scarcely no studies analyzing the influence of antiplatelet treatment on the platelet NO production. Madajka et al. showed, that ASA improves platelet NO synthesis, without significant effect on the NO bioavailability in endothelial cells [35]. According to other authors, the ASA administration seems to have two different effects on the NOS activity. Kane et al. showed that chronic acetylsalicylic acid treatment and the use of other non-steroidal anti-inflammatory drugs decrease the platelet NO production by limiting the NOS-activating response to stimulation of platelet beta-adrenergic receptors (cyclooxygenase inhibition-dependent mechanism). Nevertheless acute ASA administration activates basal platelet NOS by its acetylation and thereby acts through a mechanism independent of cyclooxygenase inhibition [36,37]. In our study platelet arachidonic acid and collagen-1—dependent aggregation was decreased on the 3rd and the 7th day after the ASA treatment onset, while the ADP-dependent aggregation was unchanged. We have also shown that during acute ASA administration arachidonic acid-induced aggregation was negatively associated with L-Arginine and the $\frac{L-Arg\;\left(PLT\right)}{ADMA\;\left(PLT\right)}$ ratio, while after 7 days platelet aggregation was positively associated with platelet ADMA bioavailability (described as high $\frac{ADMA\;\left(PLT\right)}{ADMA\;\left(PLS\right)}$ ratio) in arachidonic acid-, collagen-1 and ADP- dependent mechanism. We confirmed observations of other authors, that ASA induces the platelet NO production leading to thrombus dissolving only during acute administration, while thrombus formation in the course of chronic acetylsalicylic acid treatment is probably positively associated with ADMA in platelets to ADMA in plasma ratio. However, it is hard to distinguish between the influence of ASA treatment and the natural course of ischemic stroke disease on changes in NO biotransformation metabolites and platelet aggregation, as ASA was not administrated in the control group.

### 4.5. Demography and Comorbidity Differences between the Stroke and the Control Group—Possible Effect on the Results

Although the study protocol assumed that stroke subjects and healthy controls were closely matched, significant differences in comorbidities, drug treatment and biochemic results were found. Higher hypertension burden and angiotensin-converting-enzyme inhibitors (ACE-I) intake was detected in the stroke group in comparison to the control. Administration of some other hypotensive drugs (angiotensin-receptor blocker (ARB) β-blocker, dihydropyridine calcium channel blocker, thiazide/thiazide like diuretic, loop diuretic) was significantly higher only in the stroke group at discharge. Finally, the diagnosis of dyslipidaemia or use of statin was significantly higher at the discharge than on admission in the stroke group. Other authors have already shown that basal platelet concentration of L-Arginine and platelet derived NO production is diminished in presence of such cardiovascular risk factors as age, smoking, hypercholesterolemia with oxidized LDL, hypertension, diabetes mellitus and coronary artery disease. [28,38,39,40,41,42]. While Gryglewski et al. have shown in animal model study, that ACE-I treatment could induce thrombolysis by the increase in endothelial nitric oxide production. Moreover, statin-related improvement in the stroke outcome is documented to be mediated among others by the increase in the platelet eNOS expression [35,43,44]. Similarly increase in the L-Arginine concentration in stroke patients observed in our study could result not only from ASA but also from other drugs used during hospitalization. Nevertheless, further studies are required to verify if observed L-Arginine increase is due to the natural course of disease or whether it results from medications and from which ones.

The study group and the control group were also significantly different regarding the white blood cells count (WBC), mean platelet volume (MPV), serum glucose, potassium, and thyroid-stimulating hormone (TSH) levels. Increased WBC, together with hyperglycaemia observed in the stroke group, might be a symptom of acute stress responses involving the activation of the hypothalamic-pituitary-adrenal axis and the sympathetic nervous system in reaction to extensive brain injury. Leucocytosis is also associated with activation of coagulation cascades, thrombus formation, and is a typical finding in acute phase of ischemic stroke (thrombo-inflammation theory) [45,46]. The MPV was smaller in the stroke group, and it increased gradually in the course of disease. Although elevated MPV is a well-recognised risk factor for ischemic stroke, low MPV is a characteristic feature for patients with active thrombosis. Observed increase in MPV in the course of disease can be a response to the thrombus formation by enhanced production of antiaggregatory molecules in platelets and probably inhibition of megacariopoiesis (the greater magnitude of thrombopoiesis, the smaller platelet volume) [47,48,49,50]. Lower level of potassium in the study group corresponds to the findings of other authors who described hypokalaemia in up to ¼ of patients with ischemic stroke. One of the pathomechanisms aimed at explaining this correlation is that hypokalemia by reducing conductance hyperpolarization in potassium channel of cells promotes formation of free radicals, which could lead to endothelial dysfunction—documented ischemic stroke risk factor [51,52]. Finally, we have found higher level of TSH in the study group in comparison to the control group. Manolis et al. have already described a positive correlation between subclinical thyroid dysfunction (subclinical hypothyroidism or subclinical hyperthyroidism) and increased cardiovascular risk. However, due to lack of randomized controlled trials, no consensus has been reached on whether treatment of dysfunction is beneficial in prevention of ischemic stroke [53].

### 4.6. Perspectives

There are only few studies analyzing the influence of increased NO bioavailability on the course of ischemic stroke. Saleh et al. showed in an animal model that L-Arginine administration is more effective than ASA supplementation in primary prevention of thrombotic events, which could be achieved by greater inhibition of platelet aggregation and higher reduction of the low-density lipoprotein (LDL) oxidation in comparison to ASA treatment [54,55]. Li et al. documented in an animal model of acute ischemic stroke that platelet membrane biomimetic magnetic nanocarriers with NO achieve rapid targeting to ischemic stroke lesions, encouraging the release of L-Arginine at the thrombus site leading to disruption of the local platelet aggregation and reperfusion of the ischemic penumbra [56]. ASA combination with NO-donor could be a promising drug in prevention and treatment of non-cardioembolic ischemic stroke, but further human studies should be conducted. Moreover, studies analyzing the dynamic changes in production of platelet nitric oxide correlated with NOS expression (both endothelial and inducible one), oxidative stress parameters, and ischemic stroke outcome would be of great importance for further understanding of ischemic stroke patomechanisms, as NO not only acts as inhibitor of aggregation and vasodilator but can also be transformed to the one of reactive oxygen species.

## 5. Conclusions

Human subjects with non-cardioembolic ischemic stroke are characterized by stably elevated platelet levels of the NOS inhibitors which could be associated with increased platelet susceptibility to aggregation. ASA treatment in acute phase of stroke results in increase in the platelet L-Arginine concentration together with higher platelet NO bioavailability. It suggests that ASA has not only the cyclooxygenase-1-, but also the nitric oxide-dependent antiplatelet function. Hence, we suggest that the platelet nitric oxide and its biotransformation metabolites play an important role in regulating the aggregation in acute phase of non-cardioembolic ischemic stroke. However, further studies on platelet nitric oxide role in pathogenesis of ischemic stroke are required, aiming at explanation if decreased platelet NO bioavailability could be considered as a risk factor and simultaneously therapeutic target in ischemic stroke.

## 6. Limitations

Several limitations of this study should be underlined. The first regards selection of the stroke group, due to study inclusion and exclusion criteria. Approximately 70% of patients from the initial cohort met the exclusion criteria, as treatment by thrombolysis/thrombectomy or both could affect the measurement of nitric oxide biotransformation and platelet aggregation in the natural course of the disease. Moreover, 15% of patients had to be excluded, due to the stroke severity (lack of informed consent). Finally, only 10% of patients (with mild to moderate course of disease) from over 400 ones met the inclusion criteria which could create “parapatric speciation” bias in the study group. Another limitation regards the measured molecules. As NO is highly reactive, it was not possible to measure it directly in our experimental setting. The cyclic guanosine monophosphate (cGMP), as effector of nitric oxide but also of various other metabolic pathways, was not studied either. Hence, our study is based on more stable nitric oxide biotransformation metabolites, but in consequence it makes our conclusions on NO correlation with platelet aggregation indirect. Moreover, different permeability of the platelet membrane and laboratory separation processes could affect the inter-compartment distribution and study results. Therefore, additional experiments should be conducted in order to assess the significance of these phenomena. Finally, it is not possible to certainly distinguish between natural change in platelet function during ischemic stroke course and ASA administration effect on platelet nitric oxide production, as the control group did not receive any ASA treatment at all.

## Figures and Tables

**Table 1:** Demographic and biochemical characteristics between studied groups including cardiovascular risk stratification parameters. Results are presented as mean ± SEM if the distribution of variables were normal or median with 1st and 4th quartile if the distribution of variables were not normal.

		Stroke Group N = 40			Control Group N = 39			*p*
		Mean	[±SEM]	[1st–4th Quartile]	Mean	[±SEM]	[1st–4th Quartile]	
**Women**	**Number [%]**	18 [45%]			21 [54%]			ns
**Age**	**[y]**	63.45	±1.37		63.67	±1.65		ns
**Hemoglobin**	**[g/dL]**	14.40	±0.24		13.93	±0.32		ns
**Hematocrit**	**[%]**	42.6	±0.65		41.43	±0.9		
**RBC**	**[mln/µL]**	4.78	±0.08		4.77	±0.11		ns
**WBC 1st day**	**[k/µL]**	8.92	±0.49		6.74	±0.39		*p* < 0.05
**WBC 3rd day**	**[k/µL]**	8.82	±0.45					*p* < 0.05
**WBC 7th day**	**[k/µL]**	8.34	±0.68					ns
**PLT**	**[k/µL]**	225.79	±9.08		244.58	±9.22		ns
**MPV 1st day**	**[fl]**	9.83	±0.24		10.94	±0.17		*p* < 0.05
**MPV 3rd day**	**[fl]**	10.17	±0.30					ns
**MPV 7th day**	**[fl]**	10.20	±0.39					ns
**hsCRP**	**[mg/L]**	5.05		1.6–6.0	4.55		2.91–4.12	ns
**ESR**	**[mm/h]**	13.63		7.0–16.5	16.39		10.0–18.0	ns
**Sodium**	**[mmol/L]**	139.40	±0.41		140.19	±0.48		ns
**Potassium 1st day**	**[mmol/L]**	3.91	±0.06		4.14	±0.08		*p* < 0.05
**Potassium 3rd day**	**[mmol/L]**	3.99	±0.05					ns
**Potassium 7th day**	**[mmol/L]**	4.01	±0.09					ns
**Glucose 1st day**	**[mg/dL]**	132.97		9.8–145.0	101.12		86.0–102.0	*p* < 0.05
**Glucose 3rd day**	**[mg/dL]**	98.72		81.0–99.0				ns
**Glucose 7th day**	**[mg/dL]**	100.52		80.0–125.0				ns
**Urea**	**[mg/dL]**	33.08		26.0–38.0	32.20		25.5–36.0	ns
**Creatinine**	**[mg/dL]**	0.93		0.76–1.14	0.89		0.67–0.99	ns
**Total protein**	**[g/dL]**	6.51	±0.17		6.36	±0.14		ns
**AST**	**[IU/L]**	19.38		16.0–22.0	19.15		14.5–21.5	ns
**ALT**	**[IU/L]**	21.50		15.0–26.0	23.96		16.0–30.0	ns
**Total bilirubin**	**[mg/dL]**	0.80	±0.09		0.73	±0.07		ns
**TCh**	**[mg/dL]**	181.59	±8.08		203.38	±11.74		ns
**HDL**	**[mg/dL]**	48.18	±2.06		56.57	±3.73		ns
**LDL**	**[mg/dL]**	106.21	±6.86		122.81	±10.35		ns
**Tg**	**[mg/dL]**	128.16		91.0–142.0	128.24		87.0–163.0	ns
**TSH**	**[µIU/L]**	3.37		1.26–5.02	1.50		0.83–1.83	*p* < 0.05
**APTT**	**[s]**	27.31	±0.43		27.76	±0.64		ns
**INR**		0.99		0.94–1.04	1.00		0.96–1.03	ns

*Abbreviations*: mean platelet volume (MPV), hsCRP (high sensitivity C-reactive protein), erythrocyte sedimentation rate (ESR), total cholesterol (TCh), high-density lipoprotein (HDL), low-density lipoprotein (LDL), triglycerides (Tg), activated partial thromboplastin time (APTT), international normalized ratio (INR), ns–non significant.

## Data Availability

Data is contained within the article and supplementary material.

## Supplementary Materials

The following supporting information can be downloaded at: https://www.mdpi.com/article/10.3390/antiox11050955/s1. Table S1. Comorbidities in the control group, stroke group on admission (diagnosed before the onset of stroke) and stroke group at the discharge diagnosed both, during hospitalizationd and before onset of stroke). Table S2. Treatment applied in the control group (chronic management), stroke group on admission (until hospitalization) and stroke group at discharge. Table S3a. The correlations between NO biotransformation metabolites and platelet aggregation. Table S3b. The correlations between NO biotransformation metabolites and platelet aggregation. Table S3c. The correlations between NO biotransformation metabolites and platelet aggregation.
