# Supplementary material for: Changes in the Plasma and Platelet Nitric Oxide Biotransformation Metabolites during Ischemic Stroke—A Dynamic Human LC/MS Metabolomic Study
Source: Antioxidants (Basel). 2022 May 12;11(5):955. doi: 10.3390/antiox11050955 (PMC9137966; doi:10.3390/antiox11050955)
Supplement: Supplementary file 1 [file antioxidants-11-00955-s001.zip › antioxidants-1698706-supplementary.pdf]

Supplementary Table S1. Comorbidities in the control group, stroke group on admission (diagnosed before the onset of stroke) and stroke group at the discharge diagnosed both, during hospitalizationd and before onset of stroke).

	**Control group N=39**	**Stroke group on admission N=40**	**Stroke group at discharge N=40**	***p* value**
**Variables, units**	Mean ± SEM	Mean ± SEM	Mean ± SEM	
**BMI, kg/m2**	28.3 ± 5.6	29.7 ± 5.1	N/A	N/A
**Cigarette smoking never/ previous/ current**	37/1/1	33/3/4	N/A	N/A
**Hypertension**	18	28	35	<0.05 [a, b, c]
**Diabetes mellitus**	5	5	7	ns
**Dyslipidemia or use of statins**	17	21	40	<0.05 [b, c]
**Atrial fibrilation/flutter**	0	0	0	N/A
**Previous coronary revascularization**	0	0	0	N/A
**Previous myocardial infarction**	0	0	0	N/A
**Heart failure**	0	0	1	ns
**Peripheral artery disease**	0	0	0	N/A
**Previous stroke/TIA**	0	0	0	N/A
**Chronic kidney disease with eGFR<45ml/min.**	0	0	0	N/A
**Asthma/COPD**	1	2	2	ns

Abbreviations: SEM - standard error of mean, BMI - body mass index, TIA -transient ischemic attack, COPD- Chronic obstructive pulmonary disease, ns- non-significant, N/A – not-applicable, *p* [a] – *p*-value control group vs. stroke group on admission, *p* [b] – *p*-value control group vs. stroke group at discharge, *p* [c] – *p* value stroke group on admission vs. stroke group on discharge.

Supplementary Table S2. Treatment applied in the control group (chronic management), stroke group on admission (until hospitalization) and stroke group at discharge.

Group of drugs	Control group N=39	Stroke group on admission N=40	Stroke group at discharge N=40	*p* value
Acetylsalicylic acid	0	0	40	<0.05 [b,c]
The second antiplatelet drug	0	0	0	N/A
LMWH	0	0	0	N/A
Alteplase	0	0	0	N/A
VKA	0	0	0	N/A
NOAC	0	0	0	N/A
ACE-I	10	17	23	<0.05 [a,b,c]
ARB	7	11	16	<0.05 [b,c]
MRA	0	0	1	ns
Sacubitril/valsartan	0	0	1	ns
β-blocker	4	7	9	0.05 [b]
Digitalis glycoside	0	0	0	N/A
Calcium channel blocker (non-dihydropiridines)	0	0	0	N/A
Calcium channel blocker (dihydropiridines)	5	19	24	<0.05 [b]
α-adrenergic blocker	1	0	0	ns
Amiodarone	0	0	0	N/A
Thiazide or thiazide-like diuretic	6	8	18	<0.05 [b,c]
Loop diuretic	1	2	4	0.05 [b]
Statin	14	17	40	<0.05 [b,c]
Insulin	0	0	1	ns
Metformin	4	5	6	ns
SGLT2 inhibitor	2	2	3	ns
Oral antidiabetics other than SGLT2 inhibitor and metformin	0	1	0	ns
Proton pump inhibitor	3	2	5	0.05 [c]
Antiinflammatory drugs/NSAID/ Corticosteroids/ Immunosuppressants	0/0/0/0	0/0/0/0	0/0/0/0	N/A

Abbreviations: VKA- Vitamin K antagonist, LMWH- *Low Molecular Weight Heparin, NOAC- new oral anticoagulant, MRA- mineralocorticoid receptor antagonist, SGLT2 inhibitor-* sodium-glucose transport protein 2 inhibitor, ns- non-significant, NSAID- non-steroidal anti-inflammatory drugs, N/A – not-applicable, p^a – *p*-value control group vs. stroke group on admission, p^b – *p*-value control group vs. stroke group at discharge, p^c – *p* value stroke group on admission vs. stroke group on discharge.

Supplementary Table S3a. The correlations between NO biotransformation metabolites and platelet aggregation.

	L-Arg 1 (PLT)	L-Arg 3 (PLT)	L-Arg 7 (PLT)	L-Arg-1 (PLT)/ ADMA-1 (PLT)	L-Arg-3 (PLT)/ ADMA-3 (PLT)	L-Arg-7 (PLT)/ ADMA-7 (PLT)
**AA-1**	0.03	−0.11	−0.03	0.06	−0.03	−0.09
**AA-3**	−0.44	−0.44	−0.16	−0.38	−0.39	−0.47
**AA-7**	−0.36	−0.37	−0.20	−0.32	−0.31	−0.50
**Col-1 -1**	0.14	−0.10	−0.02	0.17	−0.01	−0.22
**Col-1 -3**	−0.04	−0.23	−0.14	0.02	−0.12	−0.50
**Col-1 -7**	−0.15	−0.29	−0.07	−0.09	−0.18	−0.67
**ADP-1**	0.10	0.00	−0.07	0.02	−0.01	−0.14
**ADP-3**	0.11	−0.03	0.02	0.04	−0.06	−0.21
**ADP-7**	0.05	−0.05	−0.08	−0.02	−0.08	−0.34

***r* ≤ (−0.01)** negative correlation; $p < 0.05$ ***r* ≥ 0.01** positive correlation; $p < 0.05$

r no significant correlation; $p > 0.05$

Abbreviations: L-Arg-1 (PLT)= L-Arginine in platelets on the 1st day, L-Arg -3 (PLT)= L-Arginine in platelets on the 3rd day, L-Arg -7 (PLT)= L-Arginine in platelets on the 7th day, L-Arg -1 (PLT)/ ADMA-1 (PLT)= L-Arginine in platelets on the 1st day to asymmetric dimethylarginine in platelets on the 1st day ration, L-Arg -3 (PLT)/ ADMA-3 (PLT))= L-Arginine in platelets on the 3rd day to asymmetric dimethylarginine in platelets on the 3rd day ratio, L-Arg -7 (PLT)/ ADMA-7 (PLT))= L-Arginine in platelets on the 7th day to asymmetric dimethylarginine in platelets on the 7tg day ratio. AA-1= arachidonic acid induced aggregation on the 1st day, AA-3= arachidonic acid induced aggregation on the 3rd day, AA-7= arachidonic acid induced aggregation on the 7th day, Col-1 -1= collagen 1μm induced aggregation on the 1st day, Col-1 -3= collagen 1μm induced aggregation on the 3rd day, Col-1 -7= collagen 1μm induced aggregation on the 7th day, ADP-1= adenosine diphosphate induced aggregation on the 1st day, ADP-3= adenosine diphosphate induced aggregation on the 3rd day, ADP-7= adenosine diphosphate induced aggregation on the 7th.

Supplementary Table S3b. The correlations between NO biotransformation metabolites and platelet aggregation.

	**Cytrulline-1 (PLT)**	**Cytrulline-3 (PLT)**	**Cytrulline-7 (PLT)**	**Ornithine-1 (PLT)**	**Ornithine-3 (PLT)**	**Ornithine-7 (PLT)**
**AA-1**	−0.15	−0.34	−0.16	−0.16	−0.35	−0.01
**AA-3**	−0.29	−0.32	−0.12	−0.19	−0.11	0.34
**AA-7**	−0.23	−0.27	−0.20	−0.13	−0.08	0.33
**Col-1 -1**	−0.10	−0.33	−0.21	0.04	−0.24	0.05
**Col-1 -3**	−0.16	−0.38	−0.24	0.06	−0.16	0.31
**Col-1 -7**	−0.19	−0.42	−0.24	−0.01	−0.21	**0.53**
**ADP-1**	−0.02	−0.04	−0.16	0.08	−0.04	0.05
**ADP-3**	0.06	−0.06	−0.09	0.18	0.08	0.16
**ADP-7**	0.01	−0.07	−0.18	0.14	0.07	0.24

$r \leq (-0.01)$ negative correlation; $p < 0.05$ **$r \geq 0.01$** positive correlation; $p < 0.05$

r no correlation; $p > 0.05$

Abbreviations: citrulline-1 (PLT) = citrulline in platelets on the 1st day; citrulline-3 (PLT) = citrulline in platelets on the 3rd day; citrulline-7 (PLT) = citrulline in platelets on the 7th day; ornithine-1 (PLT) = ornithine in platelets on the 1st day; ornithine -3 (PLT) = ornithine in platelets on the 3rd day, ornithine -7 (PLT) = ornithine in platelets on the 7th day; AA-1= arachidonic acid induced aggregation on the 1st day, AA-3= arachidonic acid induced aggregation on the 3rd day, AA-7= arachidonic acid induced aggregation on the 7th day, , Col-1 -1= collagen 1μm induced aggregation on the 1st day, Col-1 -3= collagen 1μm induced aggregation on the 3rd day, Col-1 -7= collagen 1μm induced aggregation on the 7th day, ADP-1= adenosine diphosphate induced aggregation on the 1st day, ADP-3= adenosine diphosphate induced aggregation on the 3rd day, ADP-7= adenosine diphosphate induced aggregation on the 7th day.

Supplementary Table S3c. The correlations between NO biotransformation metabolites and platelet aggregation.

	**L-Arg-1 (PLT)/ L-Arg-1 (PLS)**	**L-Arg-3 (PLT)/ L-Arg-3 (PLS)**	**L-Arg-7 (PLT)/ L-Arg-7 (PLS)**	**ADMA-1 (PLT)/ ADMA-1 (PLS)**	**ADMA-3 (PLT)/ ADMA-3 (PLS)**	**ADMA-7 (PLT)/ ADMA-7 (PLS)**
**AA-1**	0.04	0.06	0.17	0.00	−0.03	0.01
**AA-3**	−0.30	−0.24	−0.29	−0.21	−0.12	0.35
**AA-7**	−0.22	−0.17	−0.34	−0.05	−0.08	**0.39**
**Col-1 -1**	0.25	0.15	0.08	0.07	−0.10	−0.03
**Col-1 -3**	0.10	0.03	−0.21	0.02	−0.18	0.24
**Col-1 -7**	0.07	−0.06	**−0.42**	0.07	−0.23	**0.52**
**ADP-1**	0.32	039	0.08	**0.36**	0.22	0.10
**ADP-3**	0.36	**0.40**	−0.03	0.37	0.23	0.28
**ADP-7**	0.29	0.35	−0.18	0.41	0.23	0.37

$r \le (-0.01)$ negative correlation; $p < 0.05$ **$r \ge 0.01$** positive correlation; $p < 0.05$

r no significant correlation; $p > 0.05$

Abbreviations: L-Arg -1 (PLT)/ARG-1 (PLS)= L-Arginine in platelets on the 1st day to L-Arginine in plasma on the 1st day ration, L-Arg -3 (PLT)/ L-Arg -3 (PLS))= L-Arginine in platelets on the 3rd day to L-Arginine in plasma on the 3rd day ratio, L-Arg -7 (PLT)/ L-Arg -7 (PLS))= L-Arginine in platelets on the 7th day to L-Arg in plasma on the 7tg day ratio, ADMA-1 (PLT)/ADMA-1 (PLS)= asymmetric dimethylarginine in platelets on the 1st day to asymmetric dimethylarginine in plasma on the 1st day ration, ADMA-3 (PLT)/ ADMA-3 (PLS))= asymmetric dimethylarginine in platelets on the 3rd day to asymmetric dimethylarginine in plasma on the 3rd day ratio, ADMA-7 (PLT)/ ADMA-7 (PLS))= asymmetric dimethylarginine in platelets on the 7th day to asymmetric dimethylarginine in plasma on the 7tg day ratio, AA-1= arachidonic acid induced aggregation on the 1st day, AA-3= arachidonic acid induced aggregation on the 3rd day, AA-7= arachidonic acid induced aggregation on the 7th day, , Col-1 -1= collagen 1µm induced aggregation on the 1st day, Col-1 -3= collagen 1µm induced aggregation on the 3rd day, Col-1 -7= collagen 1µm induced aggregation on the 7th day, ADP-1= adenosine diphosphate induced aggregation on the 1st day, ADP-3= adenosine diphosphate induced aggregation on the 3rd day, ADP-7= adenosine diphosphate induced aggregation on the 7th day.
